# Book Review: Socio-Economic Impacts of Bioenergy Production

**DOI:** 10.3389/fbioe.2015.00174

**Published:** 2015-10-26

**Authors:** Sheikh Adil Edrisi, P. C. Abhilash

**Affiliations:** ^1^Institute of Environment and Sustainable Development, Banaras Hindu University, Varanasi, India

**Keywords:** biomass and biofuel, socio-economic factors, sustainable development, waste land development, job opportunities, income effect

During the last few decades, the bioenergy production has been radically promoted worldwide (EIA, 2013) as a clean and eco-friendly source of energy mainly due to the global concern of climate change, primarily attributed by the increasing emission of CO_2_ by fossil fuel consumptions. Although vegetable oils was first used as liquid fuels (bioenergy) in internal combustion engine by Rudolf Diesel in early 1900 (Pousa et al., 2007), the low cost and easy availability have made the fossil fuels, such as petroleum, as a primary fuel for vehicular transports. Nevertheless, the limited reserves of petroleum and its derivative had necessitated the search for cleaner and alternative source of energy (Parente, 2003; Pousa et al., 2007). Although bioenergy is considered as a versatile type of renewable energy, the large scale production of bioenergy from biomass is always under criticism as it requires large tracks of arable lands for bioenergy plantation. Hence, there is a direct conflict of interest between the food and biofuel production in the concerned societies. As a result, the present global bioenergy production is not promising and (55 EJ) (World Bioenergy Association, 2014) is still not enough to satisfy the energy demands of burgeoning global population. Hence, it is the need of the hour to maximize the bioenergy production without having any social and economic conflicts. However, the socio-economic considerations are mainly influenced by the local and regional frameworks (societal and economic) of the concerned nations, including the educational level, cultural aspects, and policies, including environmental and social targets. In this context, the book “*Socio-economic impacts of bioenergy production*” is a relevant and in-depth analysis of the socio-economic issues associated with bioenergy production and provides suitable indicators for assessing the sustainability of such bioenergy production programs and also providing strategies for overcoming all such negative impacts in an amicable way. As editors rightly pointed out, the book “*illustrates the complexity of interrelated topics in the bioenergy value chain, ranging from agriculture to conversion processes, as well as from social implications to environmental effects. It furthermore gives an outlook on future challenges associated with the expected boom of a global bio-based economy, which contributes to the paradigm shift from a fossil based to a biomass and renewable energy based economy*.” Therefore, the book is targeted to a wider readers ranging from “policy makers, scientists, and NGOs in the fields of agriculture, forestry, biotechnology, and energy.”

Though there are few books in the same domain, such as “*Sustainable Bioenergy Production: An Integrated Approach*” (Ruppert et al., 2013) and “*Bioenergy for Sustainable Development in Africa*” (Janssen and Rutz, 2012), addressing one or another aspects of socio-economic implications of bioenergy production, the current book provides a complete deliberations on the above issues along with solutions in a doable way. Therefore, we could appropriately say that the current book has its own perspectives, ideas, and deliberations that handle the cross cutting edges of socio-economic impacts of bioenergy production. Importantly, “*this publication builds upon the results of the Global-Bio-Pact project on ‘Global Assessment of Biomass and Bio-product Impacts on Socio-economics and Sustainability’ which was supported by the European Commission in the 7th Framework Programme for Research and Technological Development from February 2012 to January 2013*.” Moreover, the “*contributions to this book are based on the experience of selected authors from Europe, Africa, Asia, and Latin America, including researchers, investors, policy makers and other stakeholders such as representatives from NGOs*.”

The book basically focuses on the following aspects, such as (i) various tools for socio-economic impact assessments, (ii) indicators for assessing socio-economic sustainability, (iii) test auditing of the indicators, (iv) linkages between socio-economic and environmental impacts of bioenergy, (v) socio-economic impact of biofuels on land use change, (vi) effects on food security, (vii) socio-economic impacts of sweet sorghum value chains in temperate and tropical regions, (viii) the use of soybean by-products as a biofuel in Argentina, (ix) socio-economic impacts of palm oil and biodiesel products in Indonesia, (x) socio-economic experiences of Jatropha production in Africa and Mali, (xi) socio-economic impacts of bioethanol from sugarcane in Brazil and Costa Rica, (xii) socio-economic analysis of lignocelluloses ethanol refinery in Canada, (xiii) biogas production from organic waste in Africa, (xiv) socio-economic indicators on different bioenergy case studies, and (xv) the contribution of bioenergy to energy access and energy security.

The editors presented the intended themes in an organized and progressive manner via its arrayed, interrelated, and well-structured chapters. Furthermore, it reveals that the socio-economic impact assessment basically consists of scoping and determination of issues, social and economic baselines, its impacts, significance, mitigation, management, and monitoring. Moreover, it also describes that these assessments can also be “*used as an add-on to environmental impact assessment and/or to support biomass certification schemes*.” The introductory chapter delivers a conceptual framework regarding the different tools used for the assessment of socio-economic impacts with glorious glimpses of sustainability concepts.

The book also highlights various issues related to the impacts of bioenergy production, its assessment and screening strategies, including key indicators for test auditing and also provides linkages between the socio-economic and environmental impacts of bioenergy production and utilization. Editors have made a special attempt to address the further implications of bioenergy production on land use change and food security, but fails to explore the issues under the current global land use scenarios as the concerned chapter is only having the basic definitions of several land use pattern. However, there are some interesting sections, such as “land use rights, land tenure, and ownership,” which are useful for owing the ownership right to various stakeholders. Moreover, the impact analysis on food security is a well-handled issue corroborated with connections and controversies of food and fuel production, envisioning methodology for an economy-wide assessment of food security and biofuels, and also quantified the different biofuel policies’ impact on food security. The chapter on the socio-economic impacts of sweet sorghum is well-supported through value-chain analysis ranging from its cultivation to conversion scenarios in tropical and temperate production systems. The book also deals with the bioenergy production from soybean, palm, and sugarcane in Argentina, Indonesia, and Brazil, respectively, in different individual chapters and also emphasized Jatropha individually with its different business models in tropical areas specifically in Africa and Mali that will certainly attracts scientists, policy makers, entrepreneurs, stakeholders, and NGOs concerned with agroforestry for sustainable bioenergy production.

Apart from the issues related with the socio-economic perspectives of biomass conversion for bioenergy production, the book also addresses various issues related to the value-chain analysis of biomass conversion technologies with special reference to sugarcane to ethanol production in Costa Rica and also separately illustrates the impacts of a refinery in Canada targeting lignocellulosic ethanol production. It clearly elucidates the socio-economics of lignocellulosic biomass supply chain in a national context with special emphasis on the forestry sector and land ownership. The section also having regional and local case studies of British Columbia that deals with the lignol technology, supply chain, products of the lignol process, pyrolysis of biomass technology group (BTG), products of the BTG process, macroeconomics in the lignocellulosic biomass chain in Canada and British Columbia, employment generation, working conditions, relevance of impacts, threshold determination, mitigation options and biomass certifications, etc.

Although the use of genetically modified products of sweet sorghum and other plant products were discussed superficially, it would be much better if separate sections or chapters with specific case studies at national, regional, or local levels. The volume also lacks the future challenges and recommendations in the lignocellulosic biomass conversion processes. Moreover, the qualities of the display items (figures and illustrations) are not good and difficult to understand. However, our overall impression is that the book is a well written “*guideline to assess the socio-economic implications of bioenergy production for scientists, practitioners, and decision makers who are interested in a biomass supply, cost-value, macro and micro-economics and value-chain perspective of bioenergy production*.”

## Author Contributions

SE and PA wrote the review.

## Conflict of Interest Statement

The authors declare that the research was conducted in the absence of any commercial or financial relationships that could be construed as a potential conflict of interest.
